# Genomic medicine implementation protocols in the PhenX Toolkit: tools for standardized data collection

**DOI:** 10.1038/s41436-021-01183-0

**Published:** 2021-05-10

**Authors:** Wendy K. Chung, Kyle Brothers, Angela Bradbury, Sirisak Chanprasert, Lori Orlando, Ali Torkamani, Heather Zierhut, Marylyn D. Ritchie, Michael Phillips, Jennifer Schoden, Deborah Maiese, Tabitha Hendershot, Carol M. Hamilton, Erin M. Ramos

**Affiliations:** 1 Departments of Pediatrics and Medicine, Columbia University, New York, NY USA; 2 Department of Pediatrics, School of Medicine, University of Louisville, Louisville, KY USA; 3 Department of Medicine, Hematology/Oncology, University of Pennsylvania, Philadelphia, PA USA; 4 Department of Medical Ethics and Health Policy, Perelman School of Medicine, University of Pennsylvania, Philadelphia, PA USA; 5 Department of Medicine, University of Washington, Seattle, WA USA; 6 Department of Medicine, Duke University School of Medicine, Duke University, Durham, NC USA; 7 Department of Integrative Structural and Computational Biology, Scripps Research, La Jolla, CA USA; 8 Department of Genetics, Cell Biology, & Development, University of Minnesota, Minneapolis, MN USA; 9 Department of Genetics, Perelman School of Medicine, University of Pennsylvania, Philadelphia, PA USA; 10 RTI International, Research Triangle Park, NC USA; 11 National Human Genome Research Institute, Bethesda, MD USA

## Abstract

**Purpose:**

The PhenX Toolkit (www.phenxtoolkit.org), an online catalog of recommended measurement protocols, facilitates cross-study analyses for research with human participants. The PhenX Steering Committee recommended genomic medicine implementation as a new research domain, with the following scope: genomic knowledge and education (both patients and providers); implementation science; changes in management and treatment; return of results; patient outcomes; and ethical, legal, and social issues (ELSI) associated with genomic research.

**Methods:**

A seven-member expert Working Group convened in October 2019 to identify well-established measurement protocols for a new genomic medicine implementation domain and used the established PhenX consensus process to select measurement protocols for inclusion in the PhenX Toolkit.

**Results:**

The Working Group recommended 15 measurement protocols for inclusion in the PhenX Toolkit, with priority given to those with empirical evidence supporting validity. Consortia funded by the National Institutes of Health, and particularly the National Human Genome Research Institute, proved critical in identifying protocols with established utility in this research domain, and identified protocols that were developed through a rigorous process for scope elements that lacked formally validated protocols.

**Conclusion:**

Use of these protocols, which were released in September 2020, can facilitate standard data collection for genomic medicine implementation research.

## INTRODUCTION

Genomic medicine includes genetic and genomic testing used to stratify risk of future disease and diagnose current symptoms to enable patient and providers to make more informed decisions about prevention and treatment. The genomic medicine research community can benefit from the use of common, validated instruments to allow for comparison and aggregation of data across studies. The genomic medicine implementation (GMI) domain in the PhenX Toolkit (www.phenxtoolkit.org) comprises 15 measurement protocols that may be used to elicit knowledge of genomics, evaluate communication strategies for disclosing genetic test results, and assess genomic medicine programs.

The objective of PhenX (consensus measures for phenotypes and exposures) is to identify and promote the use of standard measurement protocols that improve the consistency of data collection and allow for cross-study analyses and increased statistical power. PhenX, which began in 2007, is funded by the National Human Genome Research Institute (NHGRI), with additional funding from other National Institutes of Health (NIH) institutes and centers. Measurement protocols are selected by Working Groups (WGs) of experts, vetted via consultation with the broader community, and made available to the public via the PhenX Toolkit (www.phenxtoolkit.org).^1,2^ The PhenX Toolkit currently includes more than 860 measurement protocols in 28 research domains and six collections with additional depth for research in substance abuse and addiction, mental health, tobacco regulatory, blood sciences, social determinants of health, and COVID-19. PhenX measurement protocols are available as Research Electronic Data Capture (REDCap) data dictionaries that can be uploaded directly to REDCap for electronic data collection. The PhenX Toolkit currently has more than 3,500 registered users and has been recommended in more than 400 NIH funding opportunities and notices and cited in 337 publications.

## MATERIALS AND METHODS

The PhenX Steering Committee (SC) developed an initial scope for a GMI domain that guided the selection of a seven-member WG. The WG members were selected to reflect the depth and diversity of the genetics and genomics field, including by discipline. The members include medical and molecular geneticists, a genetic counselor, a medical oncologist, a genome informatician, an implementation scientist, and a pediatrician/bioethicist. The WG was guided in its deliberations by an SC liaison who is a statistical and computational geneticist and an NIH project scientist who oversees the PhenX effort.

The PhenX consensus process includes defined criteria for inclusion of a measurement protocol in the PhenX Toolkit.^1^ The protocols must be well established, preferably open source, and low burden to both investigators and participants. The goal of this process is to identify protocols that are recommended and used by experts and nonexperts alike. In October 2019, the WG convened, outlined the scope of the domain (Table 1), and recommended protocols for each element of the scope.

**Table 1:** Scope of the genomic medicine implementation domain.

Return of results—to providers
Return of results—information seeking
Return of results—family communication
Implementation science—baseline
Implementation science—follow-up
Genomic knowledge—patient and provider
Change in management and treatment
Ethical, legal, and social implications (ELSI)
Patient outcomes

The PhenX consensus process also includes an email outreach that invites members of the broader scientific community to review and provide feedback on protocols being considered by a WG.

In May–June 2020, the WG solicited feedback from the PhenX listserv of subscribers and NHGRI Genomic Medicine–related consortia. Nearly 200 individuals visited the protocols for review, and the feedback provided was carefully considered by the WG before making final selections. The protocols were reviewed and approved by the PhenX SC and added to the PhenX Toolkit in September 2020. As with other PhenX research domains, the GMI protocols will be reviewed periodically to determine if each GMI protocol should remain as is, replaced with another protocol, or retired from the PhenX Toolkit. This ensures that this domain remains scientifically relevant and up to date.

## RESULTS

After recommending 18 measurement protocols for scientific community outreach, the WG identified 15 protocols for the GMI domain in the PhenX Toolkit (see Table 2).

**Table 2:** Protocols in the genomic medicine implementation domain in the PhenX Toolkit.

Protocol name	Protocol source	Description of measurement protocol	Number of items; mode of administration	Languages available; study population^a^
Adoption of Genetic Services: Health-care Settings	Reach Effectiveness Adoption Implementation Maintenance (RE-AIM) Checklist for Study or Intervention Planning^4^	The adoption dimension of RE-AIM includes a four-item checklist used to evaluate the characteristics of settings that participate in offering genetic services or a new genetic services intervention.	4 items; program evaluation	English; health-care providers
Awareness of Pharmacogenomics	Daud et al.^5^	This series of questions can be used to determine an individual’s knowledge of pharmacogenomics (PGx) and if the individual is willing to participate in PGx research. It is beneficial for patients to understand that genetic traits may influence the effects of medications and that clinicians are using PGx for patient safety purposes.	17 items; self-administered questionnaire	Dutch and English; adults, age 18 and older, and pregnant women
Baseline Knowledge of Genomics	University of North Carolina Genomic Knowledge Scale (UNC-GKS)^6^	The University of North Carolina Genomic Knowledge Scale (UNC-GKS) was developed to assess knowledge domains thought to be critical for making informed decisions about undergoing genomic sequencing (e.g., for medical diagnosis, public health applications, or guiding treatment decision making), comprehending the meaning and limitations of results, and taking appropriate actions upon learning results.	25 items; self-administered questionnaire	English; adolescents and adults age 17 and older
Clinician Confidence in Returning Genetic Test Results	Clinical Sequencing Evidence-Generating Research Consortium (CSER), Provider Measures—Post–Return of Results Follow-up #1, Electronic Medical Records and Genomics (eMERGE) Post–Return of Results Provider Follow-up Questionnaire^7^	The health-care provider is asked about his or her knowledge of genetic results without seeking information from other sources. A scale with responses from “not at all confident” to “very confident” is used to measure the provider’s confidence. A second set of items refers to the usefulness of the genetic test results with a scale that ranges from strongly disagree to strongly agree.	9 items; self-administered or interviewer-administered questionnaire	English; health-care providers after sharing the genetic results with the patient
Information Sources for Patients After Return of Results	Information Seeking V2 from the Clinical Sequencing Evidence-Generating Research (CSER) Adult Patient Measures—Post-Return of Results Follow-up #2 (5–7 months Post-Return of Results)^8^	After a patient receives genetic test results, the patient is asked which sources were used to find out more about the genetic test results. Then the patient records a score (using a scale ranging from 1 to 5) based on the perceived usefulness of the information from the source. The patient is also encouraged to list any websites that were helpful.	4 items; self-administered or interviewer-administered questionnaire	English; adult patients or parents/guardians of a pediatric patient after genetic testing
Organizational Readiness for Change	Organizational Readiness for Implementing Change (ORIC)^9^	Organizational Readiness for Implementing Change (ORIC) is a 12-item instrument used to determine how well employees at an organization feel they can implement the change in processes required by a proposed intervention. Each item includes a Likert scale ranging from 1 (Disagree) to 5 (Agree).	12 items; program evaluation	English; college students and health-care system employees
Patient Empowerment After Genetic Services and Counseling—Genomics Outcome Scale (GOS)	Genomics Outcome Scale (GOS)^10^	The Genomics Outcome Scale (GOS) is a 6-item version of the 24-item Genetic Counseling Outcome Scale (GCOS-24) and focuses on empowerment as a key outcome following counseling. The GOS includes a 5-point Likert scale eliciting the respondent’s agreement, ranging from strongly disagree (1) to strongly agree (5), with each statement.	6 items; self-administered questionnaire	English; 18 years and older in United Kingdom
Patient Response to Genetic Testing	Feelings About genomiC Testing Results (FACToR)^11^	The FACToR instrument is used to measure the psychosocial impact of returning genomic findings to patients in research and clinical practice. The instrument includes 12 items and 4 subscales (negative emotions, positive emotions, uncertainty, privacy concerns) with a 5-point Likert scale.	12 items; self-administered questionnaire	English; patients age 18 and older after genetic testing
Patient Satisfaction with Genetic Counseling	Genetic Counseling Satisfaction Scale (GCSS)^12^	The Genetic Counseling Satisfaction Scale (GCSS) is a 6-item, 5-point Likert scale used to assess patient satisfaction with genetic counseling.	6 items; self-administered questionnaire	English; adult women after genetic counseling
Perceived Compatibility with Current Clinical Practice	Implementing GeNomics In pracTicE (IGNITE) Preimplementation Provider Survey^13^	There are three items from the IGNITE Preimplementation Provider Survey that health-care providers can use to assess the compatibility and value of a genomic intervention at their clinical practice. Responses are based on a 5-point Likert scale ranging from strongly agree (5) to strongly disagree (1).	3 items; self-administered questionnaire	English; health-care providers
Program Evaluation: Patient Uptake of Genetic Services	Reach Effectiveness Adoption Implementation and Maintenance (RE-AIM) Checklist for Study or Intervention Planning^4^	The reach dimension of RE-AIM includes a 4-item checklist used to summarize the characteristics of patients who participate in the uptake of services. Genetic service programs or investigators are expected to report these details when describing patient uptake of genetic services.	4 items; program evaluation	English; health-care providers
Regret About Health-care Decisions	Decision Regret Scale^14^	The Decision Regret Scale is a 5-item Likert scale. Respondents read each statement and use the response options to indicate how much they agree or disagree with the statement. The scale numbers range from strongly agree (1) to strongly disagree (5).	5 items; self-administered questionnaire	English; adults with cancer or osteoporosis
Relative Advantage of a Genomic Medicine Intervention over Current Practice	Implementing GeNomics In pracTicE (IGNITE) Preimplementation Provider Survey^15^	There are two questions from the IGNITE Preimplementation Provider Survey that health-care providers can use to assess the relative advantage of a genomic intervention over their current practice. Responses are based on a 5-point Likert scale ranging from strongly agree (5) to strongly disagree (1).	2 items; self-administered questionnaire	English; health-care providers
Sharing Genomic Information with Relatives	Family Communication from the Clinical Sequencing Evidence-Generating Research (CSER) Adult Patient Measures—Post-Return of Results Follow-up #2 (5–7 months Post–Return of Results) and Electronic Medical Records and Genomics (eMERGE) Family Communication of Genetic Results^8^	This series of questions can be used to determine with whom (if anyone) the individual shared his or her genetic tests results and why it was important to share the results.	5 items; self-administered or interviewer-administered questionnaire	English; adults after genetic testing
Understanding of Health Implications of Genomics	KnowGene^16^	The KnowGene Scale is a 16-item scale administered to patients after genetic testing and/or genetic counseling to measure their understanding of the health implications of genetic testing results. The KnowGene Scale includes health implications to oneself as well as relatives. This measure covers penetrance, actionability, limitations of current technology, and monogenic inheritance patterns.	16 items; self-administered questionnaire	English; adults after genetic testing
**Supplemental Information**^b^				
Adherence to Clinical Practice Guidelines for Genomic Medicine^b^	Tier 1 Genomics Applications and Their Importance to Public Health^17^	The Centers for Disease Control and Prevention’s Office of Public Health Genomics developed a toolkit of genomic medicine resources for clinicians, public health professionals, public health agencies, advocates, and community leaders. The resources focus on the following tier 1 conditions: hereditary breast and ovarian cancer syndrome, Lynch syndrome, and familial hypercholesterolemia.	Not applicable; guidelines	English; clinicians and public health professionals
Intention to Share Genomic Information with Relatives and Others^b^	Implementing GeNomics In pracTicE (IGNITE) Patient Preimplementation Survey^18^	This question is used to determine with whom (if anyone) the individual plans to share his or her genetic tests results.	1 item; self-administered questionnaire	English; adults after genetic testing
Patient Empowerment After Genetic Services and Counseling^b^	Genetic Counseling Outcome Scale (GCOS-24)^19^	The 24-item Genetic Counseling Outcome Scale (GCOS-24) focuses on empowerment. GCOS-24 includes a 7-point Likert scale ranging from how much the individual strongly disagrees (1) to strongly agrees (7) with the statement. GCOS-24 evaluates genetic counseling and testing services.	24 items; self-administered questionnaire	English; adults after genetic testing and genetic counseling

^a^Study population described in the source.

^b^Supplemental information includes protocol(s) considered by the Working Group (WG) that were not selected for the PhenX Toolkit and additional comments from the WG.

Priority was given to protocols with empirical evidence supporting validity, although protocols that had undergone formal validation were not generally available for most of the scope elements. The efforts of consortia (funded by NIH and NHGRI) therefore proved critical in identifying measures with established utility, even if they had not yet been rigorously validated. The WG identified protocols from consortia including the Clinical Sequencing Evidence-Generating Research (CSER) Consortium, Electronic Medical Records and Genomics (eMERGE) Network, and Implementing GeNomics In pracTicE (IGNITE) Consortium. The PhenX Toolkit includes program evaluation and implementation science protocols from resources such as Reach Effectiveness Adoption Implementation Maintenance (RE-AIM) and the Consolidated Framework for Implementation Research (CFIR).

These measurement protocols address the following:

- Understanding of genetic information, including baseline knowledge and understanding of genetics and genomics, clinician and patient understanding of genetic test results (including pharmacogenetics), and the implications of the genetic test results for patient and family.
- Impact of genomic interventions on patients, patients’ families, and providers, including psychosocial impact of testing, decision satisfaction/regret, information seeking, information sharing, patient empowerment, provider assessment of the genomic intervention, and adherence to clinical guidelines.
- Preimplementation assessment of organizational characteristics and readiness to change to adopt genetic services.

For each protocol, the PhenX Toolkit provides details about how the protocol should be administered, whether any special training or resources are necessary, and any specific instructions that can contribute to the standardization of data collection. The PhenX Toolkit also lists references documenting the development and use of the protocols intended to help users understand potential limitations, such as use with diverse populations and availability in non-English languages. The Toolkit provides the tools to administer these protocols (with data dictionaries and data collection worksheets) to facilitate incorporation of the protocols into studies. In addition, PhenX protocols are available as data dictionaries in Research Electronic Data Capture (REDCap), and PhenX variables are available in the database of Genotypes and Phenotypes (dbGaP).

## DISCUSSION

Genomic medicine is a relatively new and emerging field, highlighting the importance of rigorously assessing the impact of genomic medicine interventions to guide implementation and also to develop and iteratively improve best practices. As work in this field broadens, genomic medicine research will be conducted by an increasingly diverse group of investigators, some of whom will not have expertise in assessing which measurement protocols to use to assess impact of interventions. Therefore, there was a need to evaluate and develop a multidimensional set of protocols that could be easily implemented and standardized across studies.

The WG focused on protocols that have been widely utilized and are the most robust protocols currently available. Despite initial concern that there was not yet sufficient consensus to recommend common protocols, the WG was able to identify many protocols that represent the current state of genomic medicine research. Additional topics were considered such as health-care utilization and economic impact, but were excluded because they deserve dedicated attention in future work.

Entire protocols in the GMI domain should be used as constructed when possible, but the WG recognized that all items in a protocol may not be relevant to a particular study. When possible, individual items should be used as originally written to allow for maximal comparability across studies. Use of the same protocols and associated metadata (e.g., data element dictionaries) across studies will allow for direct comparisons across interventions to support a stronger base for evidence-based practice.

The PhenX Toolkit currently provides recommended GMI protocols that will be invaluable to the growing community of genomic medicine researchers. With time and publication of additional studies, we anticipate that certain protocols will emerge as the most predictive and informative. In many genomic studies, impact of genomic testing has been minimal, and it is possible that the protocols used to assess impact have not focused on the right questions. For example, as protocols more sensitive to change in health behaviors or attitudes are developed, it will be necessary to reassess their utility. In addition, the social determinants of health protocols in the Toolkit support the GMI domain, but additional GMI protocols for specific populations may be necessary. Notably, the major gaps identified in the WG’s deliberations were protocols to address health-care utilization, cost of interventions, and economic impact. Payers and policymakers will require evidence of clinical utility and need to assess economic impact; therefore, this area is one of high priority for further development.

The protocols in the domain provide the opportunity to gather useful quantitative data. However, in many cases, rich qualitative data gathered through focus groups, semistructured interviews, and similar approaches will be needed to complement the data gathered via surveys.

### Conclusion

Use of the GMI protocols in the PhenX Toolkit will facilitate collection of comparable data across genomic medicine studies to allow for direct comparison of interventions and pooling of data across studies to increase sample sizes. Given the expense and time required to complete these studies and the desire to advance the field of genomic medicine as efficiently and as effectively as possible, use of common protocols is critical and will help realize the NHGRI Strategic Vision for implementation science.^3^

## Data Availability

Materials developed in support of this manuscript have been released in the PhenX Toolkit and are available to the public. The URL to the genomic medicine implementation protocols in PhenX Toolkit is https://www.phenxtoolkit.org/domains/view/310000.
